# Protocol to establish standards for the elements infection prevention and control programs and competencies for infection control professionals in Australian residential aged care homes

**DOI:** 10.1371/journal.pone.0319108

**Published:** 2025-02-24

**Authors:** Ramon Z. Shaban, Kate Curtis, Margaret Fry, Brendan McCormack, Deborah Parker, Deborough Macbeth, Brett G. Mitchell, Phillip L. Russo, N. Deborah Friedman, Noleen Bennett, Lucy Thompson, Jo-Ann Dalton, Kathy Dempsey, Belinda Henderson, Julie Considine, Rachel Bowes, Elise Campbell, Merrick Powell, Catherine Viengkham

**Affiliations:** 1 Susan Wakil School of Nursing and Midwifery, Faculty of Medicine and Health, The University of Sydney, Camperdown, New South Wales, Australia; 2 Sydney Infectious Diseases Institute, Faculty of Medicine and Health, The University of Sydney, Camperdown, New South Wales, Australia; 3 Research and Education Network, Western Sydney Local Health District, Westmead, New South Wales, Australia; 4 New South Wales High Consequence Infectious Disease Advisory Service, Westmead Hospital, Western Sydney Local Health District, Westmead, New South Wales, Australia; 5 Emergency Services, Illawarra Shoalhaven Local Health District, Wollongong Hospital, Wollongong, New South Wales, Australia; 6 School of Nursing and Midwifery, Faculty of Health, University of Technology Sydney, Ultimo, New South Wales, Australia; 7 Gold Coast Hospital and Health Service, Gold Coast University Hospital, Southport, Queensland, Australia; 8 School of Nursing, Avondale University, Lake Macquarie, New South Wales, Australia; 9 Central Coast Local Health District, Gosford, New South Wales, Australia; 10 School of Nursing and Midwifery, Monash University, Frankston, Victoria, Australia; 11 Victorian Healthcare Associated Infection Surveillance System (VICNISS) Coordinating Centre and Department of Infectious Diseases, University of Melbourne at the Peter Doherty Institute for Infection and Immunity, Melbourne, Victoria, Australia; 12 Department of Infectious Diseases, Melbourne Medical School, University of Melbourne, Parkville, Victoria, Australia; 13 Department of Nursing, School of Health Sciences, University of Melbourne, Parkville, Victoria, Australia; 14 United Protestant Association of New South Wales Ltd, Wahroonga, New South Wales, Australia; 15 Hardi Aged Care, Seven Hills, New South Wales, Australia; 16 Clinical Excellence Commission, St Leonards, New South Wales, Australia; 17 Queensland Infection Prevention and Control Unit, Queensland Health, Herston, Queensland, Australia; 18 School of Nursing and Midwifery and Centre for Quality and Patient Safety Research in the Centre for Health Transformation, Deakin University, Geelong, Victoria, Australia; 19 Eastern Health, Box Hill, Victoria, Australia; 20 Southern Cross Care (New South Wales & ACT), Epping, New South Wales, Australia; 21 Australia Commission on Safety and Quality in Health Care, Sydney, New South Wales, Australia; PLOS: Public Library of Science, UNITED KINGDOM OF GREAT BRITAIN AND NORTHERN IRELAND

## Abstract

The COVID-19 pandemic elucidated the importance of infection prevention and control (IPC) in residential aged care homes (RACHs), both on the health and wellbeing of its residents, and the staff and clinicians who care for them. While considerable efforts have been made in Australia to improve IPC both during and in the aftermath of the COVID-19 pandemic, many of these resources remain reliant on evidence originating from hospitals and acute healthcare settings. This research aims to establish the core minimum components that will populate standards for IPC programs and governance (Stream A) and for professional practice and competencies (Stream B) in RACHs. This research will be completed using a sequential three-phase design. In Phase 1, two integrative literature reviews will be completed to synthesise the elements of current global IPC programs and professional competencies in RACHs. In Phase 2, a qualitative analysis of IPC programs and professional practice in Australian RACHs using a combination of surveys and interviews will be completed. Finally, in Phase 3, an e-Delphi will be conducted to collate expert opinion and generate consensus on the minimum components of the IPC program and professionals who administer them in RACHs. The final standards and resources will be collaboratively designed with aged care partners, industry leaders, professional bodies and key Australian health policymakers. These standards seek to empower IPC and aged care staff, not only by ensuring that they are well-equipped with the knowledge and skills to implement effective IPC programs themselves, but also that the organisation is adequately prepared to provide the resources and governance systems.

## Introduction

The COVID-19 pandemic created a variety of challenges for residential aged care homes (RACH) in Australia, placing significant strain on staff and facilities and increasing the risk of poor health outcomes of residents. Indeed, healthcare-associated infections (HAIs) and communicable diseases result in disproportionate harm and mortality in older people. This issue is particularly challenging in RACHs, where the combination of communal living spaces, overlapping staff and residents’ high vulnerability to infection put older people at significant risk of adverse health outcomes. In the first year of the COVID-19 pandemic, residents of RACHs comprised less than 0.01% of cases yet accounted for over 25% of total deaths [1]. Even before COVID-19, national surveys showed that 44.7% of RACHs had experienced an influenza outbreak and 31% a gastroenteritis outbreak in the past year, and that 12% of these outbreaks were associated with at least one fatality [2]. The pandemic served as a stark reminder of the importance of effective infection prevention and control (IPC) strategies and has since paved way for major reforms in IPC requirements for RACHs in Australia.

In general terms, effective IPC in most health settings require three fundamental components:

1. An IPC program—comprising formally documented and coordinated structures, policies and processes for implementing and evaluating infection control strategies [3];
2. An infection control professional (ICP) – a qualified individual who is responsible for the development, delivery and review of the IPC program; and
3. Appropriate systems of governance that support both the program and the professional [4].

Evidence to support these components originated from the landmark 1980 study by the Centers for Disease Control and Prevention (CDC) in the United States, known as the Study on the Efficacy of Nosocomial Infection Control or ‘SENIC Project’ [5]. This study identified that hospitals with organised surveillance and control activities, an infection control nurse per 250 beds, an infection control physician, and a system for reporting infection rates to practicing surgeons had HAI rates 32% lower than hospitals without [5]. The project founded an evidence base upon which many contemporary IPC principles and practices in hospitals and acute care settings have been adapted and subsequently implemented through national guidelines [5,6].

Similar approaches to IPC have been adopted by RACHs in Australia, which now mandate the appointment of a designated infection control lead in all homes [7]. However, RACHs currently lack context-specific national standards for IPC programs, which has led to considerable variation in their practice and evaluation. A 2018 survey found that 88% of Australian RACHs reported having a designated ICP and 97.9% reported having an IPC program, including dedicated IPC plans for common infections like influenza and gastroenteritis, and for IPC practices, such as vaccination, reporting and staff training [2,8,9]. However, this survey also identified considerable heterogeneity across RACHs in the contents of their IPC programs, with the proportion of RACHs delivering IPC education programs ranging from 94% for hand hygiene, to less than 60% for the practices like respiratory hygiene, cough etiquette, aseptic technique and antimicrobial stewardship [9,10]. This same heterogeneity also applied to both the scope of practice and the formal training and qualification of the infection control staff responsible. Of the RACHs that reported having designated IPC personnel, less than half of those personnel had formal training or qualifications in IPC [8,10].

Current IPC standards for RACHs have largely been derived from evidence in acute healthcare settings and the transferability and applicability of these skills to aged care has had little systematic evaluation undertaken. In 2024, the Australian Commission of Safety and Quality in Healthcare published the *Aged Care Infection Prevention and Control Guide* [11] to support implementation of the strengthened Aged Care Quality Standards [12]. This Guide supplements the *Australian Guidelines for the Prevention and Control of Infection in Healthcare* and aims to support the aged care workforce and those providing care for older people to understand basic IPC principles and to apply them using a risk-based approach. Importantly, the Guide notes that the “core components of an IPC system in aged care settings outlined in this Guide are based on information from the *Australian Guidelines for the Prevention and Control of Infection in Healthcare* [13] and the World Health Organization’s core components for IPC [14]” both of which are not contextualised to the aged care setting in Australia.

While resources like the *Aged Care Infection Prevention and Control Guide* [11] provided much needed guidance for the sector based on sound IPC principles in acute care, it explicitly acknowledges that RACHs are, first and foremost, long-term homes for residents, where services must balance IPC practice and the provision of clinical care with the personal goals, comfort and social needs of the residents. For IPC in aged care to be both effective and feasible, these constraints must be carefully considered, and recommendations informed by evidence developed within these contexts. Therefore, the aims of this research are to:

A. Establish core requirements for the elements and governance of IPC programs in RACHs in Australia (Stream A).
B. Establish minimum practice, capability and competency standards for IPC professionals in RACHs in Australia (Stream B).

These two aims will be addressed using three sequential study phases, whereby data collected from each phase will inform the subsequent phases:

- **Phase 1** (What do we currently know?): Two integrative reviews of international literature on A) the global elements and governance systems for IPC programs in aged care, and B) the global practice standards for IPC professionals in aged care.
- **Phase 2** (What do we currently do?): A two-step qualitative study, using surveys, document analysis and interviews, of A) the content and structure of current IPC programs and governance systems in Australian RACHs, and B) the academic and professional content of IPC training courses for aged care ICPs in Australia.
- **Phase 3** (What do we need to do?): A modified e-Delphi study to generate national consensus on A) the core requirements for IPC programs and systems of governance in Australian RACHs, and B) the minimum professional practice and competency standards for aged care ICPs.

## Materials and methods

### Phase 1–integrative literature review

#### Objectives.

A. Synthesise existing recommendations and evidence that inform the elements and governance systems of IPC programs in RACHs globally.
B. Synthesise the professional practice and competency standards for RACH infection control professionals globally.

#### Study design.

Two independent integrative literature reviews of international literature will be conducted for (A) the elements and governance systems of IPC programs in RACHs, and (B) the practice and competency standards for ICPs in RACHs. An integrative literature review was selected as the review anticipates the collection data from a diverse range of sources, including both experimental and non-experimental articles. The review process will be guided by the established five step method for conducting integrative literature reviews, broadly described by Lubbe et al. (2020) and other authors [15–19].

#### Review search strategy and selection criteria.

Searches for both research questions will be conducted in multiple databases, including CINAHL, Scopus, Ovid MEDLINE, Overton and Embase. Articles must be written in English, published from 1980 and provide a qualitative review or description of the contents of IPC programs, practice and competency standards in RACHs. Articles pertaining to practice in in-hospital geriatric wards, outpatient clinics and community-based aged care will be excluded. The search will not be limited to peer-reviewed literature, as secondary sources and grey literature such as formal standards set by national organisations or professional bodies for ICPs, also contain information pertinent to the review. These sources will be identified through a combination of manual searching and citation chaining of reference lists and websites. The broad search terms for both Streams are listed in Table 1.

Additional restrictions and filters will be applied following the execution of the initial search strategy, should a database return too many irrelevant articles. If the search returns too few peer-reviewed publications that address the literature review question, subsequent searching of grey literature, including international IPC websites and publications linked to their associations, will be performed. This integrative review will enable the context of the broader issue to be explored in the absence of published literature [20,21].

**Table 1:** Search terms for the integrative literature reviews.

	Review A: Infection Control Programs	Review B: Infection Control Professionals
**Population**	N/A	‘professional’, ‘practitioner’, ‘nurse’, ‘healthcare worker’ ‘long term care’
**Intervention**	‘infection prevention’, ‘infection control’, ‘program’, ‘standard’, ‘guideline’, ‘polic*’, ‘practice’	‘infection prevention’, ‘infection control’, ‘practice’, ‘standard’, ‘regulation’, ‘guideline’
**Outcome**	‘healthcare associated infection’, ‘infection’	‘competenc*’,‘qualification’, ‘training’, ‘education’
**Setting**	‘nursing homes’, ‘homes for the aged’, ‘health services for the aged’, ‘residential aged care’, ‘long term care’	‘nursing homes’, ‘homes for the aged’, ‘health services for the aged’, ‘residential aged care’, ‘long term care’

#### Screening and data extraction.

Articles will be imported into the online platform *Covidence* [22] (Covidence, Australia, covidence.org) and independently screened for inclusion based on their title and abstract by two reviewers. Both reviewers will be experienced researchers with expertise in infection control in aged care settings. An initial pilot screening of a random 10% of articles will be completed to ensure interrater consistency. Articles with unclear or conflicting relevance will undergo a full-text review. Any remaining conflicts after the full-text review will be resolved through group discussion, and any thereafter by a third reviewer (RZS). The inclusion and exclusion process will be illustrated in a PRISMA flowchart [15–19,23], and the full list of excluded articles (and reasons for exclusion) will be made available in the appendix of the final review.

A data extraction framework will be adapted from Jones et al. (2015) [21], and include at minimum the following headings: author(s), title, publication year, article type, study type (review, survey), edition number (if applicable), country (and state), number of domains or competencies, governing body (if applicable), review date(s), accessibility. Data will also be extracted and charted by the same two reviewers who completed the screening process, with the same process used for cross-checking and conflict management.

#### Data analysis and synthesis.

A comparative analysis will be conducted in *NVivo*^*TM*^ v14 where common themes and domains for programs and practice standards are compared across countries [21]. A narrative summary of the results will be constructed, with data categorised according to recurring themes, and the variations and commonalities between literature will be summarised. Descriptive statistics in the form of frequency counts and percentages were used to provide an overview of included studies.

### Phase 2–surveys & document analysis

#### Objectives.

A. Review the elements and governance systems that comprise current IPC programs in Australian RACHs.
B. Review the contents, characteristics and learning outcomes of all current academic and professional IPC courses in Australian RACHs.

#### Study design.

Two methods of qualitative data collection and analysis will be employed in Phase 2.

- **Phase 2 (Surveys & Document Analysis):** For Stream A, a document analysis of IPC programs and governance systems in Australian RACHs will be completed based on written documents collected via an online survey. For Stream B, a document analysis of the content of current IPC courses available in Australia will be performed using publicly available curricula retrieved from institutional and university websites.
- **Phase 2 (Key Informant Interviews):** In both streams, this will be followed by interviews with key informants who are experts in the relevant areas. For IPC programs and governance, interviews will be conducted with aged care IPC leads and facility managers. For IPC courses, interviews will be conducted with the course convenors or an equivalent party responsible for the development and delivery of the course content.

#### Participants and data sources.

Data pertaining to aged care IPC programs and governance systems will be obtained directly from aged care providers and their designated IPC leads via an online survey hosted on *REDCap*^*TM*^ – a secure web-based application for data capture managed and maintained in a secure server by the University of Sydney [24,25]. Eligible participants include all currently or recently employed (within the last 12 months) IPC leads working in an operating RACH in Australia. This also includes managers responsible for IPC who may operate under other titles (e.g., clinical manager, clinical care consultant, quality control coordinator). As of 2023, there are 2,639 operating RACHs in Australia. To obtain a representative sample, proportionate stratified sampling will recruit participants based on select RACH characteristics, including size, organisation type (i.e., government, private, not-for-profit, faith-based), single or multi-site providers, jurisdiction and geographical remoteness. The target sample is > 10% of total ICPs, assuming at least one ICP employed per RACH (n =  264). Respondents will be reimbursed with an AU$15 electronic voucher for submitting a completed survey.

Data pertaining to IPC courses will be obtained from publicly sourced leaflets, promotional materials and institutional and/or university websites [26]. To be included in the study, the course must be given by an Australian registered training organisation (RTO) and deliver at least one dedicated IPC unit that includes a component targeting practice in RACHs.

#### Data collection.

For Stream A, eligible IPC leads will receive an email invitation containing the participant information package and a *REDCap*^*TM*^ survey link. Contact details for aged care providers will be obtained from a database operated by the Australian Government. Participants will be asked to confirm they have read and understood the participant information statement prior to commencing and informed consent will be implied through the submission of the online survey.

In the survey, participants will be asked to describe the contents and elements of their organisation’s IPC programs, as well as any relevant systems of governance that exist to support the program. Participants will also be able to submit copies of their organisation’s written IPC program and policy documents to support their responses. A brief demographic survey will also collect information pertaining to the RACH’s characteristics, as well as the IPC experience and role of the respondent.

For Stream B, data pertaining to the contents, structure and learning outcomes of IPC educational courses will be retrieved by researchers from publicly available university and institutional websites.

#### Data analysis.

Two data collection templates will be developed jointly by members of the research team based on the findings of Phase 1 and previous studies [26], which will inform the primary domains. For example, the template for the IPC courses will include categories such as demographics, course enrolment, fee arrangements, graduate outcomes, course content, course delivery, assessments and clinical practice requirements. Data will be extracted by the same two reviewers from Phase 1, who will also complete an initial pilot on three random data samples per source to ensure consistency in data entry [27]. Data will be analysed using *NVivo*^*TM*^ v14 [28] and directed content analysis [27,29]. The data collection template will serve as a pre-determined coding framework as specific data will be extracted into established categories. Descriptive statistics will be used to summarise the frequencies of content items. Findings will inform the interview guide for the key informants [29].

### Phase 2–Key informant interviews

#### Participants.

Data will be obtained from two populations:

- **Aged care IPC leads.** All IPC leads who meet the eligibility criteria for Phase 2 (Surveys & Document Analysis) will be eligible for the interviews. To obtain a representative sample, the target sample size for the interview is > 10% of the Phase 2 survey respondents (n =  26). Participants will first be convenience sampled from the survey respondents. If the target sample size is not reached following the recruitment of survey respondents, then additional invitations will be distributed to the full eligibility pool.
- **IPC course convenors.** The course conveyors of the IPC courses will be identified from relevant heads of schools (or equivalent) in addition to university/institution websites. Individual emails will be sent to the heads of school at each institution to obtain permission to contact the IPC course conveyor, or a member of staff they believe to be appropriate. The target sample size is 10 participants, which will account for most well-recognised courses available in Australia.

#### Data collection.

Participants will be invited via email, which will include the participant information package, including a digital consent form, and an online interview booking form. Participants will be asked to sign and return the digital consent form via email prior to the interview. Interviews will be conducted on *Microsoft Teams* [30] (Microsoft Corporation, Redmond, WA, USA), be 60 minutes in length and will be audio recorded and transcribed verbatim. The same researchers responsible for the review of Phase 1 and Phase 2 (Surveys & Document Analysis) data will complete the interviews. The researcher will explain the interview process to participants prior to the commencement of the interview, which will include the purpose of the interview, the expected duration, reimbursement, and the audio recording of the interview. If no written consent form is received, then verbal confirmation of consent will be obtained prior to commencing the interview and recorded by the interviewer.

The interviews will follow a semi-structured interview guide, which will be developed based on previous research [26], and integrated with the findings of Phases 1 and 2 (Surveys & Document Analysis). The interview will comprise open-ended questions to explore participants’ perspectives on current elements of IPC program (Stream A) or IPC courses (Stream B) and focus on the areas that require further clarification following the Phase 2 (Surveys & Document Analysis). This interview stage seeks to minimise bias, ensuring that the researcher does not make inferences based on potentially incomplete or unclear data following the document analysis.

Participants will be reimbursed with an AU$20 electronic gift voucher for completing the interview. Participants will be asked to provide or confirm their preferred email for receiving the voucher to the interviewer after interview completion.

#### Data analysis.

All transcripts will be analysed by two reviewers using *NVivo*^*TM*^ v14 [28] and the five-step framework approach [26]. Reviewers will familiarise themselves with the interviews to enable the identification of recurring themes and key ideas [31,32]. A thematic framework will then be developed jointly by the reviewers, against which all transcripts will then be systematically indexed and coded [33,34]. Once coded, interview transcripts will be examined for patterns, themes and subthemes. The findings from the interviews will be integrated with those of Phase 2 (Surveys & Document Analysis) [26,35,36].

For Stream A, Phase 2 will provide a comprehensive overview of the content and scope of current systems of IPC practice and education for RACHs in Australia. The studies will identify key components of IPC programs and governance systems that are common and consistent across aged care facilities. The outcomes will also allow for the comparison of these components with existing national IPC guidelines for traditional healthcare settings to identify actions, strategies, considerations or other features that may have uniquely emerged in the aged care setting. For Stream B, similar comparisons will be made regarding the components of IPC courses and educational programs. Furthermore, the interviews with relevant stakeholders will contribute greater richness to the data, especially the contextual and practical facilitators and barriers that affect the practice and governance of IPC in RACHs. Findings from Phase 2 will inform the items of the e-Delphi surveys in Phase 3.

### Phase 3–e-Delphi

#### Objectives.

A. Establish the core minimum requirements for the elements and governance systems of IPC programs in Australian RACHs.
B. Establish the core minimum professional skills and competencies to underpin ICP practice standards in Australian RACHs.

#### Study design.

A modified electronic-Delphi (e-Delphi) study will be used to synthesise and generate expert consensus on the minimum elements of IPC programs (Stream A) and the minimum competencies for professional IPC practice standards (Stream B) [37]. This modified e-Delphi will combine the functionality of real-time consensus with iterative survey rounds. Typically, 2 – 4 Delphi rounds are expected to achieve consensus. This method has been previously employed by researchers to identify research priorities for emergency nursing in Australia [3], establish practice standards for graduate emergency nursing programs [38], and adapt an acute nursing framework for RACHs [39].

#### Participants and eligibility criteria.

The expert panel will be convenience sampled evenly from the following populations:

- **Experts in IPC, including credentialled ICPs.** Individuals must hold a current full membership to the Australasian College for Infection Prevention and Control (ACIPC); and be involved in the practice of IPC in a clinical, academic or research setting; and be responsible for clinical, educational, research and management activities related to IPC.
- **RACH IPC leads.** Individuals currently employed as a designated IPC lead within a RACH or have been employed within the last 12 months.
- **RACH managers.** Individuals currently holding a managerial position within an aged care organisation or have held this position within the last 12 months; and have been responsible for overseeing matters pertaining to IPC in this position.

There are no specific exclusion criteria. Key stakeholder participants may decline to participate in interviews or to complete the survey.

#### Sample size.

A sample size of 364 for the Delphi has been calculated and adjusted using the method to estimate sample size in finite populations shown in Table 2. The calculation assumes a total ACIPC full membership population of 1,425 [40], an estimated IPC lead population of 2,639 and an estimated RACH manager population of 2,639 [41].

**Table 2:** Sample size calculations for the Delphi expert panel.

Assumptions	Sample Size Calculation
Z = 1.96, p = 0.5, CI = 0.05 ACIPC Membership = 1,425 RACH Managers = 2,639 RACH IPC Leads = 2,639 Total Population (*Pop*) = 6,703	$SampleSize=\frac{Z^{2}\times p\times \left(1-p\right)}{CI^{2}}=\frac{{\left(1.96\right)}^{2}\times 0.5\times \left(1-0.5\right)}{{\left(0.05\right)}^{2}}=385$ $Adjustedsamplesize=\frac{SS}{1+\frac{SS-1}{Pop}}=\frac{384}{1+\frac{384-1}{6,703}}=364$

#### Data collection.

For IPC experts, invitations and participant information packages will be issued via email through the ACIPC, which maintains the contact details for all individuals meeting the eligibility criteria as part of normal business operations. For aged care IPC leads and RACH managers, contact details for providers will be obtained from an Australian Government-operated database, and invitations will be issued by the research team. The e-Delphi will be administered electronically via the online Delphi survey software, *Surveylet* (Calibrum International, USA, calibrum.com) [42]. Participants will be asked to confirm they have read and understood the participant information statement prior to commencing, and informed consent will be implied through the submission of the online survey. Each round of the survey will be open for four weeks and reminder emails will be circulated at the end of the second and third weeks. All participants who complete a survey round will be invited to participate in the subsequent round.

The first survey round will be developed by members of the research team, comprising nurse academics, ICPs, aged care representatives and experts in the Delphi methodology [3,38]. Survey items in the first round will be informed by the outcomes of Phases 1 and 2. The survey will include three sections: (i) a demographic questionnaire, (ii) governance and content of IPC programs in RACHs, and (iii) practice and competency standards for Australian IPC professionals in RACHs. Each survey round will include Likert scale questions where experts rate the importance of statements against a four-point Likert scale (1 =  not important, 4 =  very important) [43]. Optional open-ended questions will be included with each item to allow experts to provide additional commentary. The demographic survey will capture the expert’s age, IPC experience, professional role, academic qualifications and location. If the expert is currently (or recently) employed at a RACH, the survey will also capture the characteristics of the RACH, including size, organisation type, remoteness, location, cultural and linguistic case-mix, and the administration of specialised care units (e.g., dementia).

The electronic nature of the Delphi via *Surveylet* enables experts to complete the survey and revise their responses freely until the survey closes. Panel members will be able to anonymously see other expert’s responses in real time. This information will include the distribution of panel members’ votes and comments to open-ended questions. On survey close, a summary of responses from that round will be provided to all participating experts via email prior to the start of the subsequent survey round.

Participants will be reimbursed with an AU$20 electronic gift voucher for each survey they complete. The gift voucher will be provided via email. Participants will be asked to provide their preferred email for receiving the voucher in a separate form following the completion of the survey round.

#### Data analysis.

Sample characteristics and data from each round will be summarised using descriptive statistics. Quantitative data will be imported into *SPSS v26* [44] to calculate the frequency, median, interquartile range and content validity index for each Likert scale item. Sample and demographic characteristics will also be interrogated to identify any notable variations in outcomes. Comments from open-ended questions will be analysed in *NVivo v14* [28] by two reviewers using content analysis. Feedback from qualitative data will inform the modification of items that do not reach consensus in the subsequent round.

Consensus will be determined by the level of agreement within the panel, which will be quantified using a content validity index (CVI). A CVI will be calculated for each Likert scale item as the number of participants who give a rating of 3 (moderately important) or 4 (very important) divided by the total number of participants that responded to that question. Items that reach a CVI of 80% are considered to have reached a high level of consensus and thus accepted by the expert panel. Items that do not reach consensus will be modified based on expert feedback and forwarded to the subsequent round. Internal consistency will be determined for each round of the Delphi survey using the Cronbach alpha coefficient. Differences between item agreement in Delphi rounds will be examined using paired t-tests and χ^2^ tests. A p-value <  0.05 will be considered statistically significant.

Data from Phases 1, 2 and 3 will be integrated using thematic analysis following the framework by Braun and Clarke [45]. These outcomes will generate the evidence base to inform the first standards for IPC programs, governance and professional practice for RACHs in Australia.

### Ethical considerations

This research has been approved by the University of Sydney Human Research Ethics Committee (2024/HE000272).

#### Participant recruitment and informed consent.

Written, verbal or implied informed consent will be sought from all individuals participating in the surveys or interviews for Phase 2 and in the e-Delphi for Phase 3. For surveys, informed consent will be implied through the submission of a completed survey. Prior to starting the survey, participants will also be asked to confirm they have read and understood the participant information statement. For interviews, participants may either return a signed copy of an electronic consent form or give verbal consent to the interviewer prior to commencing the interview. Participants will have the right to request access to the information they will have provided to the research team. They will be able to access a copy of the results by direct request to the chief investigator (RZS).

#### Confidentiality and privacy.

Participant privacy will be protected by blind copying participants in email communications. No personal identifiers will be collected during the data collection. Interview transcripts (Phase 2) will be de-identified by investigators. Participants who complete the Phase 2 and/or Phase 3 surveys will be assigned unique IDs on analysis. Participants completing the Phase 3 e-Delphi will be required to enter their email address, however this will not be linked to the survey responses, and instead will be used to provide feedback on the results for the completed round, with an invitation to complete the next round where relevant.

All surveys will be completed in either *REDCap*^*TM*^ (Phase 2) or *Surveylet* (Phase 3). Once surveys close, data files will be downloaded directly from the survey tool and stored on the secure Research Data Store (RDS) maintained by the University of Sydney. The RDS is accessible only to study investigators nominated by the chief investigator. All results related to participant characteristics will be presented as aggregate data and no data that identifies individual participant(s) will be reported in publications or presentations arising from the study.

#### Safety considerations.

There are no expected risks to participants and participation is voluntary. There will be no direct contact between the research team and participants for the initial invitation in Phases 2 or 3. Subsequent contact is at the discretion of the participant by providing their email address. All data transmission and collection software used are secure, including *REDCap*^*TM*^, *Calibrum* and *Microsoft Teams* [30]. No data will be transferred to the ACIPC or used in any other capacities by third parties.

### Data management

All data will be managed in accordance with the University of Sydney’s Research Data Management Policy and Procedures. All data will be stored on password-protected confidential servers within the University of Sydney, Camperdown Campus in accordance with prevailing legislation policies at both institutions. Access to data files will be provided to approved members of the research team only by the chief investigator. Data will not be used for any additional purposes beyond those described in this study. In accordance with The University of Sydney Policy, records for this study will be stored securely in the RDS for five years following publication of the results before being securely destroyed. All data will be stored securely in the RDS at the University of Sydney for five years, except as required by law, and then securely destroyed.

### Project timeline

This project has been funded by the National Health and Medical Research Council (TCR2034723) for four years. Research activities for Phase 1 will commence in January 2025 following the recruitment of all research staff. All three research phases are scheduled to be completed within the first three years, which includes up to six months of buffer time that can be allocated as needed for preparation between the phases. For Phase 1, six months will be allocated for the completion of the integrative literature review). Following that, six months will be allocated for the completion of Phase 2 (Surveys & Document Analysis), followed by another six months for the completion of Phase 2 (Key Informant Interviews). Finally, the Phase 3 e-Delphi will be completed over 12 months, which includes time allocated to recruit of the expert panel, provide feedback between rounds and develop the survey instruments based on the outcomes of Phase 1 and 2. The fourth and final year, following the completion of Phase 3, will be dedicated to the translation and dissemination of the outcomes in the form of formal standards, guides and documentation templates, as well as peer-reviewed publications.

## Discussion

The recent COVID-19 pandemic has brought to light the importance of IPC in RACHs, both on the health and wellbeing of residents, as well as the care staff, family and clinicians who support them. This research will deliver an urgently needed evidence base to inform the standards for IPC programs, governance and professionals in RACHs. The introduction of these standards will lead to greater consistency in IPC practice in aged care by standardising the contents of IPC programs and how they are governed. For educators, this will provide a framework for the training, education and assessment of ICPs, by informing the curriculum of approved IPC courses. It will serve as a critical foundation for the position descriptions against which qualified ICPs are identified and hired, a baseline expectation for their roles and responsibilities, and key performance indicators against which they are evaluated. The generation of this new knowledge will support the development of appropriate standards which will empower IPC and aged care staff. The standards will ensure that staff and organisational leaders are well-equipped with the knowledge and skills to implement effective IPC programs themselves and knowing their organisation is adequately prepared to provide the resources and governance systems. This research will enable RACHs to strengthen their response to incoming standards, better maximise efforts to prevent and control infection and provide a safer, higher quality and more effective healthcare environment for both residents and staff.

## Limitations of the study design

This research aims to complete a comprehensive and thorough synthesis of existing empirical IPC evidence to generate formal standards for RACHs using expert consensus. However, it is well-established that most evidence currently originates from hospitals and acute healthcare settings, and empirical research specific to the RACH context remains limited. This research ultimately relies on opinion and input of experts to adapt standards that are suitable and appropriate for the aged care sector. Therefore, some recommendations may be provided in lieu of objective research evidence to support it and there is no guarantee that all recommendations in this study will be effective.

## Dissemination plan

The outcomes from each phase of the research will be disseminated in a variety of forums, including open access peer-reviewed scientific journals, conference proceedings, social media, press releases and other local, national and international presentations. All publications will include information on the sources of financial and in-kind support for the research and any potential conflicts of interest. Authorship for publications will be decided prior to each publication and will comply with the International Committee of Medical Journal Editors (ICMJE) guidelines for peer-reviewed publications [46].

## Amendments to the protocol

Major amendments or variations to the research design or methodology, including major delays or termination, will be documented and submitted to the University of Sydney HREC for approval, and where applicable to the NHMRC in accordance with grant guidelines. Minor variations that do not materially affect the conditions of the human research ethics approval, cause significant disruptions to the research, or its completion will be documented by the chief investigator in an implementation log and submitted as part of an annual progress report to the University of Sydney HREC and the NHMRC in accordance with standard operating procedures.
